# The Programmed Cell Death of Macrophages, Endothelial Cells, and Tubular Epithelial Cells in Sepsis-AKI

**DOI:** 10.3389/fmed.2021.796724

**Published:** 2021-12-02

**Authors:** Chao Li, Wei Wang, Shuai-shuai Xie, Wen-xian Ma, Qian-wen Fan, Ying Chen, Yuan He, Jia-nan Wang, Qin Yang, Hai-di Li, Juan Jin, Ming-ming Liu, Xiao-ming Meng, Jia-gen Wen

**Affiliations:** ^1^Inflammation and Immune Mediated Diseases Laboratory of Anhui Province, The Key Laboratory of Anti-Inflammatory of Immune Medicines (Ministry of Education), Anhui Institute of Innovative Drugs, School of Pharmacy, Anhui Medical University, Hefei, China; ^2^Anhui Province Key Laboratory of Genitourinary Diseases, Department of Urology and Institute of Urology, The First Affiliated Hospital of Anhui Medical University, Anhui Medical University, Hefei, China; ^3^Key Laboratory of Anti-inflammatory and Immunopharmacology (Ministry of Education), Department of Pharmacology, Anhui Medical University, Hefei, China

**Keywords:** sepsis, acute kidney injury, apoptosis, pyroptosis, necroptosis

## Abstract

Sepsis is a systemic inflammatory response syndrome caused by infection, following with acute injury to multiple organs. Sepsis-induced acute kidney injury (AKI) is currently recognized as one of the most severe complications related to sepsis. The pathophysiology of sepsis-AKI involves multiple cell types, including macrophages, vascular endothelial cells (ECs) and renal tubular epithelial cells (TECs), etc. More significantly, programmed cell death including apoptosis, necroptosis and pyroptosis could be triggered by sepsis in these types of cells, which enhances AKI progress. Moreover, the cross-talk and connections between these cells and cell death are critical for better understanding the pathophysiological basis of sepsis-AKI. Mitochondria dysfunction and oxidative stress are traditionally considered as the leading triggers of programmed cell death. Recent findings also highlight that autophagy, mitochondria quality control and epigenetic modification, which interact with programmed cell death, participate in the damage process in sepsis-AKI. The insightful understanding of the programmed cell death in sepsis-AKI could facilitate the development of effective treatment, as well as preventive methods.

## Introduction

Sepsis is a systemic inflammatory response syndrome caused by infection and is one of the leading causes of death worldwide (1–3). It is a complex disease characterized by severe systemic inflammation and multiple organ dysfunction (e.g., blood vessels, lungs, liver, kidneys, heart, and brain) due to the host's unregulated response to infection (4, 5). Multidrug-resistant bacteria, drug side effects and adverse events make the treatment of sepsis considerably complex (2, 6–9). Therefore, sepsis is a global public health challenge (10).

The damage of kidney is observed during the progression of sepsis, especially for these severe sepsis patients. However, the exact cause of this damage induced by sepsis remains unclear (11–13). According to statistics, ~50% of acute kidney injury (AKI) cases are associated with severe sepsis. Compared with patients without AKI, the risk of death in patients with sepsis and AKI increases by six to eight times, and after sepsis, the risk of AKI developing into chronic kidney disease increases by three times (14–17). However, the exact cause of this damage induced by sepsis remains unclear. The pathogenesis of sepsis-induced AKI (sepsis-AKI) is extremely complicated, including inflammation, hemodynamics, microvascular dysfunction and renal tubular damage. Currently, there is no effective treatment for sepsis-AKI other than supportive care in the form of dialysis. Despite considerable progress in this field (18–21), there are still no standardized and satisfactory therapeutic strategies for sepsis-AKI.

## Macrophages, Vascular Endothelial Cells and Renal Tubular Epithelial Cells are Involved in Sepsis-AKI

Macrophages, vascular endothelial cells (ECs) and renal tubular epithelial cells (TECs) in the kidney play an important role in the maintenance. The dysfunction or death of these cells are closely involved in sepsis-AKI (22–24). During the host's defense against infection, macrophages play a central role in innate immunity (25, 26), recognizing pathogen-associated molecular patterns (PAMPs) and/or host-derived damage-associated molecular patterns (DAMPs) through pattern recognition receptors (27, 28). PAMPs and/or DAMPs released from damaged tissues activate and promote the pro-inflammatory phenotype (M1) of macrophages, leading to the release of pro-inflammatory cytokines such as interleukin (IL)-1, IL-6, tumor necrosis factor-α (TNF-α), chemokines and reactive oxygen species (ROS), which cause damage to the kidney tissue (29, 30). The second vulnerable cell type is ECs. The distribution of renal blood vessels is complex, including the renal arteries, glomerular arterioles, glomerular capillary network, arcuate arteries, and renal capillaries. Vascular ECs cover the inside of renal blood vessels, which are involved in oxygen and nutrient transport, glomerular filtration and maintenance of microcirculation homeostasis. Current evidence has clarified that the damage and dysfunction of vascular ECs are the leading causes of acute lung injury in sepsis (31). However, there are few studies on renal vascular EC fate during sepsis. Endotoxins, DAMPs, inflammatory factors, ROS, and vasoconstrictor substances present in the plasma can cause dysfunction and apoptosis of vascular ECs, abnormalities in the contraction and relaxation of local blood vessels and formation of microthrombi, thereby resulting in the restriction of local blood supply and microcirculation disorders (32). The death of capillary ECs, destruction of the gap junction, hydrolysis and shedding of glycocalyx and VE-cadherin result in increased vascular permeability (33). Thereafter, a large amount of fluid, endotoxins and inflammatory factors permeate into the renal interstitium, and immune cells, including monocytes and neutrophils infiltrate, leading to renal oedema thus aggravating renal inflammation. Renal tissue oedema hinders the transport of oxygen to renal cells and exacerbates local hypoxia in the tissue. The infiltrating inflammatory cells and a large number of inflammatory factors attack the renal TECs that cause the deterioration of renal function. While renal TECs are stimulated with lipopolysaccharide (LPS) *in vitro*, the appearance of cell damage is unobvious. Ding et al. (34) and Yuan et al. (35) showed that cell damage appeared when the concentration of LPS reached 100 μg/ml. These evidence suggested that sepsis-AKI is a complicated disease that involves the cross-link between the macrophages, ECs and TECs.

The renal tubules and the glomerulus that are mainly composed of TECs, ECs and glomerular mesangial cells are the main components of the kidney, responsible for the renal function. Damage to the renal tubules was distinctly observed in patients and animal models with sepsis, with the appearance of a large number of renal tubular casts (36). The damaged tubules obstruct the flow of the tubular fluid and cause the renal unit to lose its filtration function. Currently, it has been suggested that the abnormal structure and function of renal tubules are the most important and direct causes of sepsis-AKI (37).

**Figure 1:**
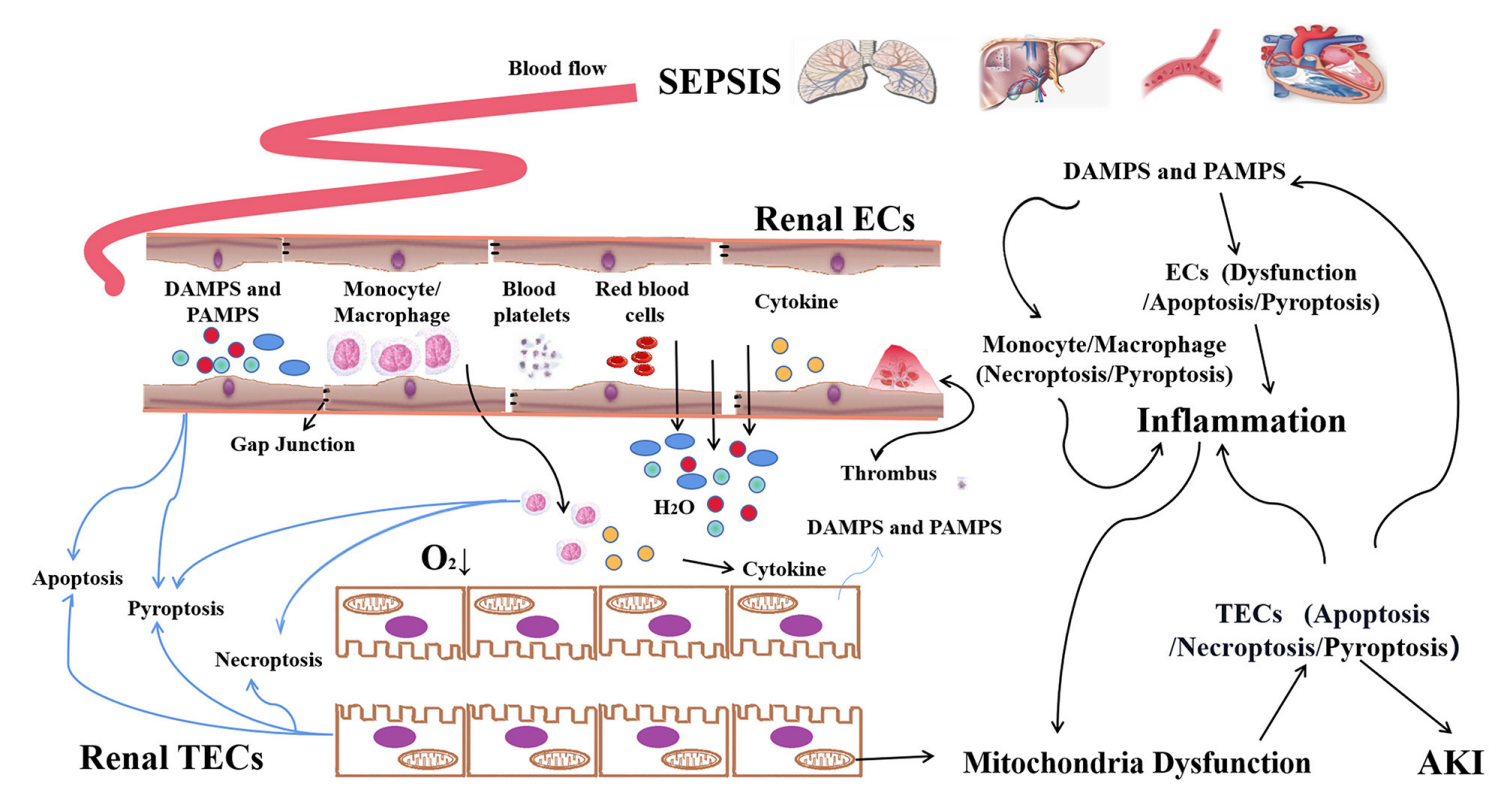
The programmed death of macrophages, ECs, and TECs in sepsis-AKI. In sepsis, PAMPs and/or DAMPs that are released from damaged tissues activate and increase the pro-inflammatory phenotype (M1) forms of macrophages, leading to the release of pro-inflammatory cytokines which can cause damage to the kidney tissue of bystanders. PAMPs/DAMPs and etc present in the plasma can induce the dysfunction and cell death of vascular ECs. Abnormalities in the contraction and relaxation of local blood vessels, as well as the formation of microthrombus, result in the restriction of local blood supply and microcirculation disorders. Apoptosis and pyroptosis of ECs result in the increasing of vascular permeability. Thereafter, a large amount of fluid, endotoxin and inflammatory factors and cells permeated into the renal interstitium, further exacerbating local hypoxia. The infiltrating inflammatory cells and inflammatory factors, as well as the ROS, attacked TECs. Finally, TECs undergo apoptosis, pyroptosis and necroptosis, leading to AKI.

In summary, the damage and dysfunction of inflammatory cells, vascular ECs and renal TECs, and their cross-talk and connections are the pathophysiological basis for the occurrence of sepsis-AKI (Figure 1). Currently, the changes and regulatory processes of these cells involved in the development of sepsis have been partially studied. This review will discuss the different types of cell death and their regulation in sepsis-AKI, as well as summarize the intervention approaches and potential therapeutic targets.

## Types of Programmed Cell Death in Sepsis-AKI

At present, the forms of cell death that are associated with AKI can be roughly divided into apoptosis, pyroptosis, necroptosis, and ferroptosis (38–42). The types of cell death in sepsis-AKI depend on cell type, microenvironment and the stage of kidney damage. The development of sepsis-AKI may involve multiple cell death pathways, and the molecular mechanisms of these pathways are different but interrelated. Therefore, in the following sections, we briefly describe the relationship between various cell death pathways in sepsis-AKI.

### Apoptosis

N. Lerolle used kidney biopsy samples from patients who died of septic shock for histological examination. Apoptotic cells were found in the proximal and distal tubules of all septic samples as indicated by TdT-mediated dUTP nick-end labeling (TUNEL) or caspase-3 staining. In addition, TUNEL-positive cells were detected in less numbers in the glomeruli (36). Similar results were obtained by Aslan et al. (43). In animal models of sepsis including those induced by either cecal ligation and puncture (CLP), LPS or bacterial-induced sepsis-AKI, apoptosis was observed in both renal tubular cells and vascular ECs (44, 45). Even in cultured TECs and vascular ECs, LPS could induce apoptosis (46). However, it is still unclear how these cells undergo apoptosis in response to various signals and stimuli. Recent evidence highlighted that the generation of ROS, dysfunction of the mitochondria, and activation of TNF-α signaling were involved in triggering apoptosis.

During sepsis, macrophages, ECs and TECs undergo apoptosis and appear as apoptotic bodies. The mechanism by which these cells initiate apoptosis upon injury remains unclear. LPS stimulation increases the levels of TNF-α and Fas ligand which initiate cell apoptosis by activating TNFR and subsequent caspase signals. In addition to TNF-α, LPS can directly induce apoptosis in bovine glomerular ECs and cultured tubular cells (47). Bannerman et al. proposed that LPS can directly aggregate Fas-associated death domain (FADD) and TNF receptor-associated death domain (TRADD) through the death effector domain (DED) to induce the activation of caspase-3, 6 and 8 (48). The activation of caspase-3 signaling results in inhibition of proteolysis and leads to irreversible cell death. Activated caspase-3 translocates into the nucleus, leading to substrate cleavage, DNA degradation and protein modification, resulting in the appearance of apoptotic bodies. In addition to TNF signal, endotoxin stimulation and/or oxygen limitation lead to the generation of ROS which can also initiate apoptosis. Excessive ROS can stimulate p53 that induces apoptosis. Moreover, B-cell lymphoma-2-associated X (Bax) is activated by ROS and/or p53 and translocated to the outer membrane of the mitochondria, leading to the leakage of cytochrome c (cyto c), which is a strong inducer of apoptosis (49). Therefore, the apoptosis signaling pathway is closely related to sepsis-AKI, and inhibiting apoptosis may be an effective strategy for the prevention and treatment of sepsis-AKI.

Immune cell infiltration is a characteristic of endotoxin-induced kidney injury, and is composed of monocytes/macrophages, neutrophils and T cells. During the development of sepsis, the depletion of macrophages hinders microbial clearance (50), and excessive apoptosis of macrophages may lead to immune suppression risk of secondary infection and even death (51). Anti-apoptotic therapy targeting macrophages by anti-apoptotic proteins, such as regulating B-cell lymphoma-2 (Bcl-2) family members, can effectively reduce the morbidity and mortality induced by sepsis (52). LPS-induced inflammation of mouse macrophages is related to the production of hydrogen sulfide (H_2_S). George et al. (53) found that the use of H_2_S inhibitors (DL-propargylglycine) can significantly reduce LPS-induced apoptosis and inflammation, and suggest that P53 and Bax may be involved in this process. Chen et al. (54) found that resveratrol inhibited pro-inflammatory macrophages, by inducing apoptosis of activated macrophages, and reducing LPS-induced renal inflammation in sepsis-AKI mice. For kidney, it may be beneficial to induce macrophage apoptosis that limits the release of pro-inflammatory factors and the activation of other immune cells.

Endothelial dysfunction and damage are considered to be the cause of multiple organ dysfunction. Studies have confirmed that the apoptosis of ECs caused by sepsis induces cardiac dysfunction in rats (55). Both Fu et al. (56) and Dong et al. (57) confirmed that the use of CLP-induced rat serum and LPS caused apoptosis of vascular ECs. Hou et al. (58) found that rat CLP induced abdominal aorta EC injury and apoptosis, characterized by nuclear pyknosis and vascular wall shedding and increased number of TUNEL-positive cells. Additionally, *in vitro* experiments showed that LPS stimulation induced ECs apoptosis, with specific manifestations of chromatin fragments and apoptotic bodies. This study also demonstrated a protective effect of adiponectin on ECs in sepsis-AKI in both *in vivo* and *in vitro* models, and suggested that adiponectin could alleviate EC apoptosis by inhibiting oxidative and endoplasmic reticulum stress (58). This evidence suggested that EC apoptosis might be mediated by LPS-induced oxidative damage. However, whether the intervening of apoptosis in ECs could prevent kidney from damage during sepsis still needs to be explored.

Recently, targeting apoptosis of TECs is thought to be a useful strategy to protect sepsis-AKI (55). The inhibiting of 15-hydroxyprostaglandin dehydrogenase, the key enzyme involved in prostaglandin degradation, can reverse the apoptosis of renal cells induced by LPS through the mitochondrial-related prostaglandin degradation pathway (59). Cell death dff45-like effector C (CIDEC) can directly down-regulate AMPK activity by interacting with AMPKα subunits, and silencing CIDEC reduces LPS-induced epithelial cell apoptosis (60). The thiourea analog SPA0355 inhibits LPS-induced apoptosis of tubule cells by inhibiting the p53 signaling pathway (61). Moreover, current studies have shown that the active ingredients of Chinese herbal medicines and their derivatives such as dihydroartemisinin (62), ginkgetin aglycone (63), geniposide (64), and neferine (65) can reduce the apoptosis of TECs and LPS-induced acute kidney injury.

### Pyroptosis

Pyroptosis caused by PAMPs, DAMPs and inflammatory factors is the most important form of programmed necrosis associated with the development of septic shock and tissue damage. It is a newly defined inflammatory cell death that is different from other forms of programmed cell death (66, 67). This type of cell death leads to further release of large amounts of DAMPs and pro-inflammatory factors, as well as the activation of inflammatory cells, thereby amplifying the inflammatory responses and the damage of various tissues (68, 69).

PAMPs and DAMPs are recognized by a variety of specialized pattern recognition receptors (PRRs) (70, 71), such as Toll-like receptor 4 (TLR4), expressed on the cell surface of phagocytes and other types of cells (72). TLR4 is highly expressed on the surfaces of both macrophages and phagocytes, and its expression is further increased during sepsis. Upon activation, TLR4 rapidly recruits the adaptor protein MyD88 which interacts with interleukin-1 receptor-associated kinase 1 (IRAK1) and TNF receptor-associated factor 6 (TRAF6) to act on a series of intermediates for downstream signal amplification and NF-κB activation (73, 74). Consequently, the expression of TNF-α, IL-1β, and other genes are increased (75). In addition, nucleoside-binding oligomer domains (NOD) and leucine-rich repeat sequence (LRR) receptors (NLRs) in the cytoplasm are also the recognition receptors of PAMPs and DAMPs (76). NLRP3 is one of the most important intracellular receptors that also can be transcriptionally induced by NF-κB, and the activation of NLRP3 inflammasomes plays a key role in pyroptosis (77–79). After recognizing the ligands, NLRP3 captured the adaptor protein apoptosis-associated speck-like protein containing a CARD (ASC) and further activated caspase-1 leading to the maturation of inflammatory factors such as IL-18 and IL-1β (80–82). Activated caspase-1 cleaves the N-terminal sequence of gasdermin D (GSDMD) to bind to the membrane and generate membrane pores, resulting in cell swelling, membrane rupture and cell death. In the non-classical pyroptosis pathway, human caspase-4, 5 and mouse caspase-11 can be directly activated upon interaction with bacterial LPS, resulting in the cleavage of GSDMD, independent of NLPR3 and caspase-1 activation (83–85).

The generation of ROS is a potential trigger for the assembly of NLRP3 inflammasomes (86, 87). Increased levels of mitochondrial ROS (mtROS) are induced due to infection, inflammation or mitochondrial dysfunction, mtDNA is released into the cytoplasm in an oxidized form. This occurs through the direct interaction of oxidized mitochondrial DNA (ox-mtDNA) with NLRP3, triggering the assembly and activation of the NLRP3 inflammasome (88–90).

In sepsis-AKI, pyroptosis is observed in the cell types of macrophages, ECs and TECs (91–93). Compared to *in vitro* experiments, *in vivo* experiments showed even more severe pyroptosis in TECs. Current evidence shows that the inhibition of pyroptosis signaling can ameliorate sepsis-AKI in mice.

M1 macrophages are polarized under the stimulation of pro-inflammatory factors, such as infection, endotoxin and hypoxia. After stimulation with LPS, the TNF-α/HMGB1 pathway in M1 macrophages is activated leading to an increase in pyroptosis. Inhibition of TNF-α signaling can significantly suppress macrophage pyroptosis (94). Both M1 and M2 macrophages participate in the process of AKI by influencing the levels of inflammatory factors and cell death, including pyroptosis. Juan et al. (95) proved that M1 and M2 macrophages have opposite effects on the pyroptosis of renal TECs in a co-culture system, which may lead to functional differences of their different phenotypes in the pathology of sepsis-AKI. Additional evidence showed that either CLP or LPS caused macrophage pyroptosis (93, 96). Activated NLRP3 forms a protein complex (NLRP3 inflammasome) consisting of NLRP3, ASC and precaspase-1, and activated caspase-1 causes macrophage pyroptosis (97). To explore how macrophages perceive intracellular LPS and function in the host at the whole body level, Kumari et al. (98) constructed a conditional knockout of caspase-11 from monocytes/macrophages, neutrophils, and dendritic cells in mice, and found that macrophage/monocyte-specific caspase-11 plays a leading role in mediating the pathological manifestations of endotoxemia, including the activation of GSDMD, IL-1β and IL-18, the release of DAMPs and tissue damage, proving that caspase-11 dependent macrophage pyroptosis is pivotal in the development of sepsis.

In LPS-activated macrophages, 4-hydroxycinnamaldehyde-galactosamine (HCAG) also reduced NLRP3 inflammasome activation and derived IL-1β secretion by inhibiting the ATP-mediated phosphorylation of AKT and PKC-α/δ. *In vivo*, HCAG inhibited the expressions of NLRP3, Caspase-1, IL-1β, IL-18, TLR4, and MyD88 in mouse renal tissue, and reduced LPS-induced renal inflammation (99). In order to find potential therapeutic drugs to inhibit macrophage pyroptosis, Zhao et al. (100) identified samotolisib (ST), a novel dual phosphoinositide 3-kinase (PI3K) and mammalian target of rapamycin (mTOR) inhibitor, exhibited the best inhibitory effect on inflammation in RAW 264.7 cells. Further molecular analysis showed that the protective mechanism of ST may mediate the expression of the ubiquitin ligase Nedd4, which binds and ubiquitinates caspase-11 through the PI3K/AKT/ mTOR signaling pathway.

In sepsis, ECs also appear in pyroptosis. Cheng et al. (101) firstly confirmed that the conditional knockout of caspase-11 in ECs can reduce endotoxin-induced pulmonary oedema, neutrophil accumulation and pyroptosis. There is also evidence that the cytoplasmic calcium signal promotes the transfer of activated GSDMD to the plasma membrane instead of increasing its expression, thereby inducing the formation of plasma membrane pores and achieving LPS-induced EC pyroptosis (102).

Recently, Zhao et al. (103) emphasized the importance of SP1/reticulocalbin-2 (RCN2)/ROS signaling pathway in the regulation of EC pyroptosis induced by LPS. RCN2 protein can be expressed in the normal arteries of mice and upregulated in arteries with structural remodeling after intimal injury, associating with the expression of endothelial inflammatory genes and endothelial nitric oxide synthase (eNOS) (104, 105). In the study, knockdown of RCN2 could antagonize the inhibitory effect of LPS on eNOS phosphorylation, confirming that RCN2-mediated EC pyroptosis depends on the production of ROS. Moreover, it was confirmed that specific protein 1 (SP1) could directly bind to the promoter region of RCN2 and regulate its transcription, suggesting the importance of SP1/RCN2/ROS signaling pathway in promoting EC pyroptosis. Using the isolated ECs from the lung of mice, Chen et al. (106) found that GSDMD was responsible for LPS-induced cell death when treated *in vitro* with LPS plus HMGB1. Reconstitution of GSDMD in GSDMD deficient cells restored the sensitivity of ECs to the cytotoxic effect of LPS.

Recent research has confirmed the high expression of the active forms of caspase-1, GSDMD and inflammatory cytokines IL-1β and IL-18 in primary human TECs and LPS-induced mouse kidneys (107). The use of caspase-1 inhibitors can reduce the expression of NLRP1 inflammasome and the pyroptosis of TECs (108). By employing genetic and pharmacological methods, targeting the pyroptosis pathway can effectively reduce the expression and function of NLRP3 and GSDMD in sepsis-AKI (107, 109–111). In addition, silencing caspase-11 in mice and TECs can also inhibit LPS-induced pyroptosis. Upon administration of a lethal dose of LPS, caspase-11 knockout mice prevented pathological abnormalities and survived (112). Thus, caspase-11 also plays a key role in the pyroptosis of TECs (113–116). More recently, Wang et al. (117) confirmed that after the onset of sepsis, TLR2 enhances the pyroptosis signal in TECs by regulating endoplasmic reticulum stress, which may be the key mechanism for triggering pyroptosis. These findings indicate that pyroptosis of TECs may be one of the main mechanisms involved in the renal damage of sepsis (107).

### Necroptosis

Programmed cell necrosis, also named neroptosis, was observed in cisplatin-, IR- and contrast- induced nephrotoxicity. The activation of receptor interacting protein kinase 1 (RIPK1), which is mainly through its combination to TNFR, is widely regarded as the main mediator of necroptosis (118). RIPK1 further activates RIPK3 and thereafter mixed lineage kinase domain-like (MLKL), *via* a homologous interaction motif (RHIM) (119–121). Upon activation, the oligomers of phosphorylated MLKL protein form and translocate to the plasma membrane. This structure can cause cell swelling, rupture and the releasing of DAMPs (122, 123). The pathophysiology of sepsis-AKI differs from that of other AKI models. Upon systemic administration of a lethal dose of LPS, no obvious necrosis of renal TECs was observed in septic kidney (124). Therefore, the role of necroptosis in sepsis-AKI has aroused the interest of current studies.

Recently, Shutinoski et al. (125) confirmed that, in the absence of caspase signal, the K45A mutation in RIPK1 leads to poor phosphorylation of RIPK1 and RIPK3 as well as reduction in MLKL trimers and macrophage necroptosis caused by LPS. RIPK1-K45A mutant mice exhibit a better anti-inflammatory response *in vivo* and have significant resistance to shock caused by endotoxins. Bone marrow transplantation confirmed that necroptosis and pyrolysis of myeloid and non-myeloid cells are indispensable for the progression of multiple organ damage caused by sepsis. In myeloid cells, RIPK3-mediated necroptosis and GSDMD-mediated pyrolysis work together during the process of sepsis and may synergistically enhance tissue inflammation and damage (106). This suggests that necroptosis, as well as pyrolysis, of macrophages plays an important role in sepsis-AKI, through the rapid release of a great mass of pro-inflammatory factors.

Overactivation of the endothelin-1 (ET-1) system contributes to endothelial dysfunction. Abdul et al. (126) found that LPS decreased endogenous ET-1 secretion, increased ET receptor expression and activated necroptosis in human brain microvascular endothelial cells (BMVECs). Blocking of ET receptors with bosentan inhibited the necroptosis pathway and improved the cell migration ability of BMVECs. In a mouse model of severe acute respiratory distress syndrome (ARDS) induced with high-dose LPS, Yu et al. (127) found that lung injury was mainly due to RIPK3-MLKL-mediated necroptosis and endothelial dysfunction. Although the research on sepsis-induced necroptosis of renal ECs is scare, the role of endothelial necroptosis should not be ignored.

For TECs, Sureshbabu et al. (128) showed that the expression levels of phosphorylated RIPK3 (p-RIPK3), RIPK3, and p-MLKL increased in a time-dependant manner in human renal proximal tubular cells (HK-2 cells) in response to LPS challenge. In mice subjected to CLP surgery, the expression levels of p-RIPK3, RIPK3, and MLKL were higher in the kidney tissues at 6 h after treatment. These results indicate that necroptosis of TECs is also involved in the occurrence and development of sepsis-AKI. However, both the necroptosis of ECs and TECs during sepsis are less investigated. Here, we proposed that the necroptosis regulation of renal vascular ECs and renal TECs should be highlighted.

### Relationship Between Apoptosis, Pyroptosis, and Necroptosis

Apoptosis, pyroptosis and necroptosis, with their unique morphologic, cell biologic, and biochemical features, are the main kinds of programmed cell death during sepsis-AKI, and other types of death including ferroptosis are seldom reported. Upon the current finding, it is becoming clear that three types of death are mutually interlinked. Previous studies have demonstrated the occurrence of both apoptosis, pyroptosis, necroptosis in sepsis-AKI. For example, tubular apoptosis and necrosis were found in the kidney tissue of sepsis-AKI. Guo et al. (129) and Liu et al. (130) found that apoptosis and pyroptosis coexist in CLP mice. These evidence mean that all these kinds of programmed cell death can appear at the same time in kideny.

In addition, apoptosis, pyroptosis and necroptosis are mutually co-influenced. There are many cross-pathway signaling events among apoptosis, pyroptosis and necroptosis. The most prominent among these is caspase-8 which interacts with the different cell death pathways. Caspase-8 is proposed to interact with ASC to activate apoptosis (131, 132) and to intensify the apoptotic cascade leading to the inhibition of necroptosis (133). The research of Fritsch et al. (134) showed that the expression of enzymatically inactive caspase-8 (C362S) causes embryonic lethality in mice, possibly because of the enhanced necroptosis and pyroptosis. MLKL deficiency rescued the cardiovascular phenotype but unexpectedly caused perinatal lethality in caspase-8^C362S/C362S^ mice, indicating that pyroptosis occurs when apoptosis and necroptosis are inhibited simultaneously (134). Moreover, both embryonic lethality and premature death were completely rescued in caspase-8^C362S/C362S^MLKL^−/−^ASC^−/−^ or caspase-8^C362S/C362S^MLKL^−/−^caspase-1^−/−^ mice, indicating that caspase-8 represents the molecular switch that controls apoptosis, necroptosis and pyroptosis (134).

However, limited information concerning the regulatonal process between these types of programmed cell death is available. Based on the nature signaling pathway, it seems probable that the mutual regulation of three types of cell death occurs at different times and development stages, as well as different types of cells, during the progression of sepsis. Understanding the interplay between different cell death pathways and elucidating the precise mechanisms behind cell death is essential to the development of potential therapies to treat sepsis-AKI.

## Cellular Processes Interact With Programmed Cell Death

Understanding and solving the sophisticated network involved in the pathogenesis of sepsis, especially the control of cell death, is one of the main challenges in intensive care medicine. Mitochondrial dysfunction, oxidative stress/nitrosative stress signals, autophagy, mitophagy, and epigenetic modification are all involved in the regulation of cell death in sepsis-AKI. These may be regulatory hinges that induce or attenuate cell injury, with the possibility of becoming therapeutic targets in treating sepsis-AKI.

### Mitochondria and Oxidative Stress/Nitrosative Stress

**Figure 2:**
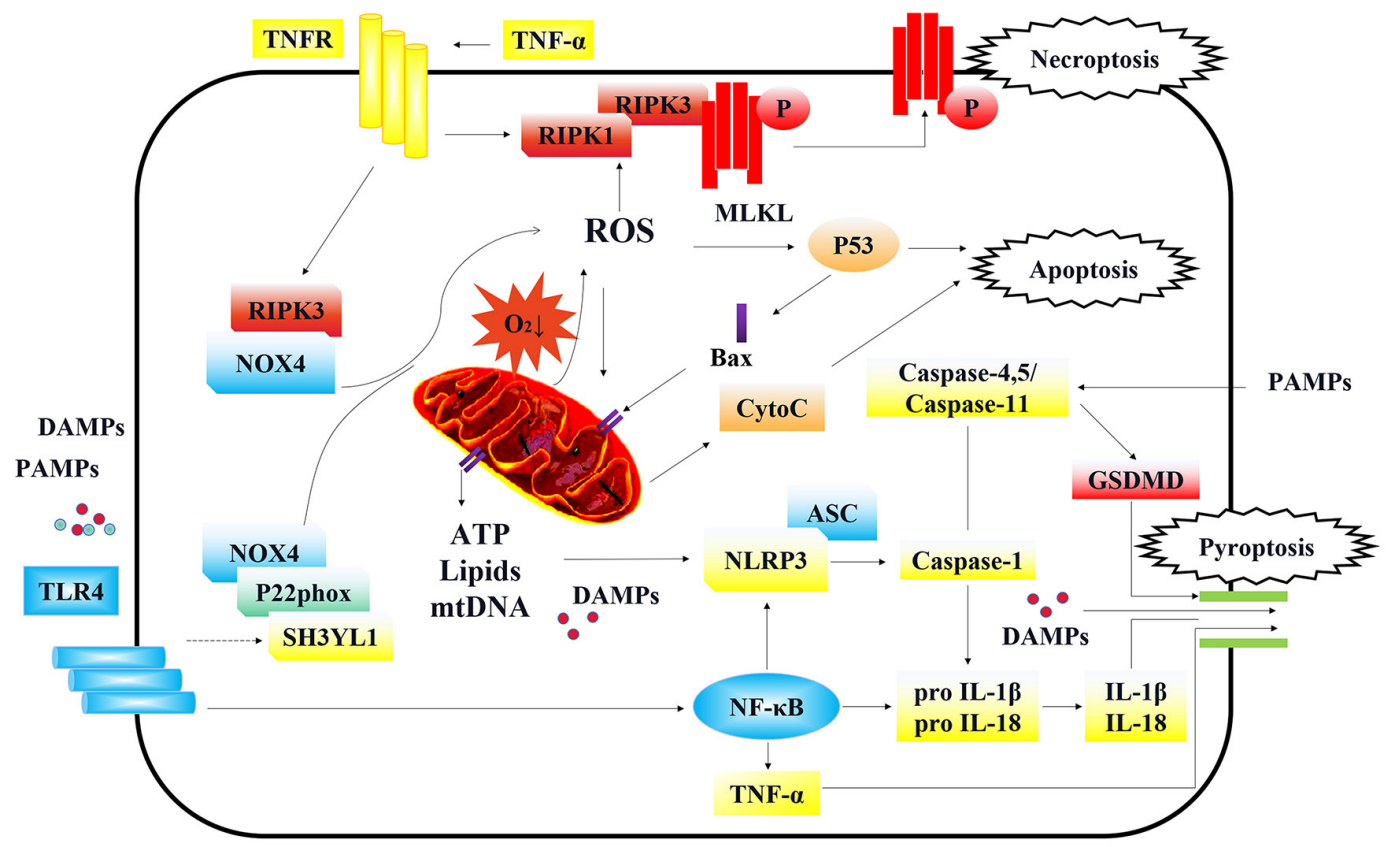
Cellular signaling in regulating programmed cell death of TECs. Under the stimulation of DAMPs/PAMPs, TLR4 is activated and initiates the downstream NOX4 signal. The enhanced connection between SH3YL1 and the anchoring protein P22phox of NOX4, stabilizes and activate NOX4, leading to the production of ROS and further mitochondrial injury. Furthermore, RIPK3 can also directly bind and stabilize NOX4 and bring it to mitochondria, causing mitochondria dysfunction. On the other hand, ROS and activated TNFR are the main inducements of necroptosis. In an oxygen-deficient environment, mitochondria dysfunction can lead to the production of ROS, which activates p53 and causes apoptosis. On the contrary, p53 further activates the formation of Bax dimers which are translocated to the outer membrane of mitochondria leading to the release of mitochondrial cyto C and other potential proinflammatory substances including membrane lipids, ATP, and oxidized form of mtDNA. The substance leaked from mitochondria, triggered the assembly and activation of the NLRP3 inflammasome, causing cell pyroptosis. Besides, NLRP3 can also be activated by the PAMPs entered through endocytosis. NLRP3 captured adaption protein ASC and further activated caspase-1 that led to maturation of inflammatory factors IL-18 and IL-1β and the cleavage of GSDMD. Moreover, TLR4 can activate the NF-κB signaling pathway which transcriptionally regulates the production of TNF-α, IL-1β, IL-18, and NLRP3. Caspase-11 in mice and its homologs in humans (caspase-4/5) can be directly activated by PAMPs, independent of NLRP3 activation. Finally, the cleaved GSDMD by caspase-1 formed the structure of membrane pores, leading to the release of pro-inflammatory cytokines, damage associated molecular patterns (DAMPs) and other cellular contents. After releasing to the extracellular environment, DAMPs and pro-inflammatory cytokines further aggravated the necroptosis and pyroptosis, in cycles.

Mitochondria play an important role in energy metabolism. The kidneys are one of the most energy-consuming organs and are rich in mitochondria. Previous studies have shown that mitochondrial dysfunction is an important factor in the pathogenesis of AKI (135). During sepsis, the renal proximal tubules are highly susceptible to an imbalance in energy metabolism. More importantly, several studies have shown that mitochondrial dysfunction of TECs is a predisposing factor for sepsis-AKI (136). Mitochondrial dysfunction is closely related to variations in the intracellular and extracellular environments. During the process of sepsis, renal microcirculation and inflammatory insults lead to abnormalities of mitochondria in both vascular ECs and TECs, causing the damage of renal cells (Figure 2) (137–139).

Under hypoxia, the type of glucose oxidative respiration changes and the levels of aerobic glycolysis increase. Meanwhile, pyruvate is converted to lactic acid under the action of enzymes, and glucose oxidative phosphorylation and electron respiration transmission chain are blocked. A large amount of pyruvate accumulates in the mitochondria and forms succinic acid. When the tissue is suddenly reoxygenated, succinic acid is rapidly oxidized, leading to the formation of excess superoxide anion (${\text{O}}_{2^{-}}$) (140–142). In addition, in the normal condition of mitochondria, the superoxide anions produced by complexes I, II, or III can be converted into H_2_O_2_ by superoxide dismutase (SOD), which is subsequently decomposed into H_2_O by catalase (CAT) (143, 144). The SOD/CAT activity ratio is very important for maintaining the levels of oxidative stress inside cells. Several studies have shown that the SOD/CAT ratio increases significantly under LPS stimulation or sepsis in multiple organs and tissues (145). The SOD/CAT ratio showed similar changes in the kidney model of sepsis. At the same time, it was also accompanied by a decline in the levels of substances and enzymes, such as the glutathione reduction system. After the production of excess ROS, cells develop a status of oxidative stress, resulting in the oxidatively modification of DNA, proteins and lipids. ROS also directly damages the structure of mitochondria and further aggravates its biosynthesis (137, 138).

In addition, the cytoplasmic levels of the NADPH oxidase family (NOX) members are also essential for ROS generation. In mouse ECs, when sepsis occurs, NOX1, NOX2 and NOX4 are significantly up-regulated. The interference of NOX1 and NOX4 by siRNA can significantly reduce the production of ROS. In addition, NOX4 interference can reduce the mortality of CLP-induced septic mice and inhibit lung inflammation (146). Geis et al. (147) reported that early expression of NOX4 was the cause of neuralgia. However, in kidney diseases, especially sepsis-AKI, there are few studies explaining the effects of NOX family members on oxidative stress and diseases. Our research found that in diabetic nephropathy and cisplatin-induced AKI, NOX4-dependant ROS significantly promoted the progression of disease (139, 148). Moreover, there is a close association between NOX4 induction and ROS production in other acute and chronic kidney diseases (149). However, in obesity-caused renal vascular endothelial damage, ${\text{O}}_{2^{-}}$ produced by NOX2 plays a more important role (150). In mouse renal TECs, Yoo et al. (151) confirmed that LPS stimulation led to enhanced interaction between SH3YL1 and the anchoring protein P22phox of NOX4, thereby further activating NOX4 and leading to oxidative damage in the cells. In a CLP-induced AKI model, Angara Sureshbabu et al. (128) found that necroptosis was activated in kidney cells, and co-immunoprecipitation (co-IP) demonstrated that RIPK3 could directly bind and stabilize NOX4, independent of its downstream signal, MLKL. Furthermore, RIPK3 could transport the complex to the mitochondria, and hence, affect the oxidative respiratory chain, aggravate oxidative stress and cause mitochondrial dysfunction.

Mitochondrial dysfunction caused by inflammation and oxidative stress is further linked to apoptosis, pyroptosis, and necroptosis (152–154). Recently, Diaz-Quintana et al. (155) reported that oxidative stress attacks cardiolipin, a lipid component on the outer membrane of the mitochondria, by oxidizing the lipids and uncoupling cyto c from cardiolipin to generate an oxidized form of cyto c. At the same time, due to the destruction of mitochondrial membrane lipids, cyto c is released into the cytoplasm and activates the caspase family, causing cell apoptosis. It has also been reported that ROS activates Bax either through the JNK/p38 mitogen-activated protein kinase (MAPK) signaling pathway or directly by phosphorylation, and activated Bax forms dimers and translocates to the mitochondria (138, 139). Additionally, ROS can cause apoptosis through activating p53 or Bim (Bcl-2 interacting mediator of cell death), inhibiting the mitochondrial protective protein Bcl-2, and further activating the Bax complex to form membrane pores outside the mitochondria, leading to the release of mitochondrial cyto c (154). At the same time, other mitochondrial contents, including damaged membrane lipids, ATP, and oxidized form of mtDNA are also released. These substances, ROS, and endotoxins that are endocytosed into cells are all activated ligands of NLRP3 (156, 157). Therefore, mitochondrial dysfunction can cause apoptosis and pyroptosis through the leakage of mitochondrial contents.

Both TECs and renal vascular ECs may also serve as therapeutic targets for mitochondrial antioxidants (141). Numerous agents targeting mitochondria and redox homeostasis, can prevent the occurrence of pathological oxidative stress and protect against sepsis-induced AKI (Table 1).

**Table 1:** The intervention drugs of programmed cell death in sepsis-AKI.

**Cell death**	**Intervention drugs**	**Mechanism**	**Model**	**References**
Apoptosis	DL-propargylglycine	Decreasing Bax, P53 and inflammation	LPS-RAW264.7	(53)
	Resveratrol	Decreasing iNOS, Bcl-2 and Bcl-xL in macrophages; inhibiting macrophages activation, cytokine release and TLR4 activation	LPS-Mice	(54)
	Adiponectin	Decreasing GRP78, CHOP and caspase-12; attenuating endoplasmic reticulum stress IRE1α pathway and ROS	CLP-Rat; LPS-HUVEC	(58)
	SW033291	Inhibiting 15-PGDH; increasing Bcl-2, downregulating Fas, caspase-3, caspase-8; downregulating lipid peroxidation and oxidative stress	LPS-Mice	(59)
	SPA0355	Inhibiting P53 signaling pathway, inflammation, oxidative stress	LPS-Mice	(61)
	Dihydroartemisinin	Inhibiting caspase-3, inflammation, oxidative stress	LPS-Mice	(62)
	Ginkgetin aglycone	Upregulating SIRT1, blocking inflammation	LPS-Mice; LPS-HK-2	(63)
	Geniposide	Activating PPARγ; increasing Bcl-2; decreasing Bax and cleaved caspase-3; reducing vascular permeability, inflammation and oxidative stress	CLP-Mice; LPS-HK-2	(64)
	Neferine	Upregulating Klotho, reducing inflammation	LPS-Mice; LPS-NRK52E	(65)
	TMP195	Inhibiting IIa HDACs, increasing Bax and cleaved caspase3, decreasing Bcl-2 and bone morphogenetic protein-7, mitigating inflammation	LPS-Mice; LPS-Murine RTEC	(189)
	Dexmedetomidine	Reducing MALAT1, ALKBH5 and inflammation	LPS-HK-2	(211)
	AICAR or metformin	Increasing Sirt3, activating AMPK, restorating mitochondrial function and metabolic fitness	CLP-Mice; LPS+HMGB1-HK-2	(212)
	Paricalcitol	Increasing vitamin D receptor and Bcl2, decreasing cleaved caspase-3	LPS-Mice	(213)
	Arbutin	Regulating PI3K/Akt/Nrf2 pathway; increasing Bcl-2; decreasing Bax, caspase-3, and caspase-9	LPS-Rat; LPS-NRK-52e	(214)
	(–)-epigallocatechin-3-gallate	Inhibiting Sema3A; increasing Bcl-2; decreasing Bax and cleaved caspase-3	LPS-Mice; LPS-NRK52E	(215)
Pyroptosis	CC-5013	Inhibiting TNF-α/HMGB1 signaling pathway and inflammation	LPS/D-Gal,mice;LPS-M1 macrophage	(94)
	HCAG	Decreasing NLRP3, caspase-1, IL-1β, IL-18, TLR4 and MyD88; inhibiting phosphorylation of AKT and PKC-α/δ	LPS-RAW264.7; LPS-Mice	(99)
	Samotolisib	Inhibiting PI3K/AKT/ mTOR pathway, Nedd4 and caspase-11	LPS-Mice; LPS-RAW264.7	(100)
	Mdivi-1	Inhibiting DLP1; reducing NLRP3, cleaved caspase-1, GSDMD, IL-1β and IL-18	LPS-Mice; LPS-Mouse RTEC	(130)
	Znpp	Inhibiting HO-1/PINK1, inflammation and oxidative stress; regulating mitochondria fusion/fission	LPS-Rat	(181)
	AC-YVAD-CMK	Inhibiting caspase-1; decreasing NLRP-1, IL-1β, IL-18 and GSDMD	CLP-Mice	(108)
	C16(C13H8N4OS)	Inhibiting protein kinase R, decreasing ASC, NLRP3, caspase-1	LPS-Mice	(216)
	Thymoquinone	Decreasing NLRP3, caspase-1, caspase-3, caspase-8, and inflammation	CLP-Mice	(129)
	N-acetyl-L-cysteine	Removing ROS, decreasing cleaved caspase-1, NLRP3, and cleaved GSDMD	LPS-HUVECs	(103)
	HU308	Increasing cannabinoid receptor 2; decreasing NLRP3, caspase-1 and GSDMD activation	CLP-Mice; LPS-BMDM	(96)
Necroptosis	Bosentan	Blocking ET receptors; decreasing TLR4, MyD88 and phosphorylation of RIPK3; improving cell migration property	LPS- BMVECs	(126)
	Necrostatin-1	Inhibiting RIPK1; decreasing LC3-II and p62	LPS-Mice	(217)
Autophagy	Resveratrol or quercetin	Activating Sirt1, deacetylating P53	CLP/LPS-Mice; LPS-RTEC	(218)
	2-DG	Inhibiting aerobic glycolysis, activating Lactic/SIRT3/AMPK pathway	CLP-Mice; LPS-HK-2	(219)
	Dexmedetomidine	Decreasing NLRP3,IL-1β and IL-18, ASC, caspase-1 and cleaved caspase-1; increasing LC3 and beclin-1;activating α2-AR/AMPK/mTOR pathway	LPS-Rat	(178)

In addition to oxidative stress, nitrosative stress is also vital for septic kidney. The source of nitrosative stress, NO, is a chemical synthesized by nitric oxide synthase (NOS), with great vasodilation ability but a short half-life (158–160). There are currently three types of NOS: nNOS, iNOS, and eNOS. eNOS was originally discovered in the endothelium, including the renal vascular endothelium. nNOS is predominantly expressed in neurons, while iNOS is constitutively expressed in both mouse and human renal tubule cells and contributes to subsequent renal haemodynamic changes and reduction in glomerular filtration rate (GFR) during the first stage of sepsis-induced AKI (161, 162). Langenberg et al. (163) demonstrated that production of all NOS isoforms was increased during sepsis in the renal cortex. NO reacts immediately with elevated levels of superoxide ions and generates peroxynitrite radicals. Peroxynitrite is a powerful oxidant capable of oxidizing thiol groups and DNA bases and modifying proteins and lipids by nitration. Furthermore, peroxynitrite directly insults the mitochondrial membrane and inhibits the respiratory chain complex, causing mitochondrial dysfunction (164). A number of animal studies have demonstrated that selective iNOS inhibition attenuates sepsis-induced renal dysfunction and improves survival (165–168). Moderate levels of nitric oxide in the renal vascular endothelium may protect the kidney by inhibiting platelet-aggregation-related glomerular microthrombi and causing cyclic GMP-mediated vasodilatation to counteract renal vasoconstriction with increased activity of the sympathetic nervous system and angiotensin II during sepsis (169, 170). Excessive production of NO in the renal vascular endothelium is also harmful, causing damage to the endothelium, affecting vascular tone and resulting in blood leakage (171, 172).

### Autophagy and Mitophagy

Autophagy is a highly conservative physiological process. In the context of intracellular damage, lysosomal degradation and recycle of damaged organelles are used to maintain cell homeostasis and remove harmful substances, which is conducive to cell survival and physiological functions (173–175). The process of autophagy mainly includes the formation of autophagosomes, the fusion of autophagosomes and lysosomes with autophagosomes, and the degradation of substrates in autophagosomes (176). Autophagy dysfunction is related to the pathogenesis of sepsis-AKI. Zhang et al. (177) found that AKI induced by LPS had self-repairing ability through autophagy. Yang et al. (178) found that the use of the anesthetic drug dexmedetomidine could enhance autophagy in LPS-insulted rat kidney, through the activation of a2-AR/AMPK/mTOR pathway. Meanwhile, the inhibition of NLRP3 inflammasome and alleviation of AKI were also observed after treatment with dexmedetomidine. The autophagy inhibitor (3-methyladenine, 3-MA) significantly diminished the protective effects of dexmedetomidine. Therefore, autophagy can protect the kidney from sepsis-AKI by inhibiting apoptosis and pyrolysis. More interestingly, it was reported that the RIPK3-related signal of necroptosis was capable of inhibiting the autophagy, in the *in vitro* and *in vivo* models of septic AKI. The study demonstrated that RIPK3 binded to transcription factor EB, which blocked the transcription factor EB-lysosomal pathway, inhibited autophagy degradation and participated in the spesis-induced renal damage (179).

Under physiological conditions, the division and fusion of mitochondria is an ongoing process, which plays a key role in mitochondrial quality control and cellular energy metabolism. Mitochondrial division separates the damaged mitochondrial part from the normal part. If the divided mitochondria are severely damaged, they undergo mitophagy, which is selective degradation of damaged mitochondria, and the degraded components are recycled and reused (180). In sepsis-AKI, a large number of mitochondria in TECs undergo fragmentation, suggesting that fission plays an important role in this pathological process. However, both *in vivo* and *in vitro* sepsis-AKI models showed that the DRP1 inhibitor Mdivi-1 suppressed mitochondrial fission, down-regulated the expression of NLRP3 inflammasome-related proteins, improved mitochondrial function, and reduced pyroptosis of TECs (130). Similar to the effect of Mdivi-1, Znpp (an HO-1 inhibitor) could reverse the mitochondrial division and pyroptosis caused by LPS. These studies suggest that in septic kidneys, excessive mitochondrial division may also have a negative effect on the fate of cells (181).

Under oxidative stress, the permeability of the mitochondrial membrane increases vigorously with a decrease in the membrane potential, so that PINK1 can stabilize on the surface of the outer mitochondrial membrane. PINK1 further recruits and binds PARKIN, leading to ubiquitination and phosphorylation of the outer membrane components, and finally, the ubiquitinated mitochondria combine with LC3 to form autophagosomes (153, 154). Furthermore, the study also found that Bcl2 family members, BNIP3 and BNIP3L, were located on the outer surface of injured mitochondria and directly interacted with LC3 to initiate autophagy (182). Mitochondrial autophagy removes damaged mitochondria, which is important for maintaining its normal structure and function. Moreover, the mitochondrial contents are enclosed in a membrane vesicle structure and subsequently undergo dissolution by lysosomal enzymes, reducing oxidative stress, and hence do not induce cell apoptosis and pyroptosis (183). Inflammatory factors of sepsis are regarded to induce a large quantity of intracellular and extracellular oxidative species. There is an observed dynamic interaction between mitochondrial fission and mitophagy levels in CLP-induced septic kidney cells, which is obvious in the early stage of CLP (within 4–12 h). However, in the late stage, mitochondrial fission and mitophagy disappear, and cell apoptosis and pyroptosis occur (184). Recently, Wang et al. (185) found that, in rat TECs and CLP models upon inhibition of PINK1 and PARKIN, mitochondrial autophagy receptors were affected, and cell apoptosis and the decline in renal function was aggravated.

### Epigenetics Modification

Epigenetics modification, a form of inheritance without genetic variation, is obervated in both eukaryotic and prokaryotic cells, including acetylation, methylation, phosphorylation, ubiquitination, SUMOylation, carbonylation, glycosylation and microRNA expression (186). The growth, development, and repair of the kidneys, including sepsis-AKI, can be regulated by epigenetic modifications.

#### Proteic Acetylation

Acetylation of the N-terminal lysine residues of histones neutralizes the positive charge, which reduces the affinity of the histones for negatively charged DNA, and subsequently changes the condensed chromatin to unstable, open, and loose staining, allowing the recruitment of gene transcription activators or inhibitors (187). This process can be reversed by the activity of histone deacetylases (HDACs) (188). This is required for embryonic development of kidney and participates in the pathology of renal diseases. Recently, Zhang et al. (189) found that TMP195, a selective class IIa HDAC inhibitor, protects the kidney by down-regulating TECs apoptosis and inflammation, suggesting that targeting class IIa HDACs may be a new treatment strategy for sepsis-AKI, which can avoid unexpected side effects of pan HDAC inhibitors.

Deacetylase Sirtuin1 (Sirt1) dependent pathway might play a pivotal role in sepsis-AKI. It was reported that activation of Sirt1 reversed the high permeability induced by LPS, and reduced the formation of stress fiber and destruction of VE-cadherin distribution. Furthermore, LPS binding to receptor for advanced glycation end-products (RAGE) is widely acknowledged, exogenous soluble RAGE could block the response to LPS with the intracellular domain of RAGE. RAGE antibody, an exogenous biological antibodies against RAGE can reduce the downregulation and ubiquitination of Sirt1 in human umbilical vein ECs exposed to LPS. This result suggests that deacetylation plays an important role in LPS-induced injury of ECs. At present, several reports have revealed that enhanced acetylation of p53 promoted apoptosis (190). As refer to sepsis, the expression of p53 protein in the renal cortex or TECs did not change significantly, but the translocation of p53 from the nucleus to the cytoplasm increased, in consistence with the enhanced acetylation of p53, which could cause cell cycle arrest and promote apoptosis. This regulated molecular process aggravated the endothelial barrier dysfunction during LPS stimulation (191). Regarding the protective mechanism associated with sepsis-AKI, both the activation of Sirt1 induced by resveratrol/quercetin and the mutation of the acetylated lysine site of p53 alleviated sepsis-AKI.

#### DNA Methylation

DNA methylation is another typical epigenetic modification that affects DNA (192). It is catalyzed by a family of DNA methyltransferases (DNMTs) that add s-adenosylmethionine (SAM-CH3) to the methyl (CH3) of cytosine or adenine DNA nucleotides. Methylation of cytosine-phosphate-guanine (CpG) dinucleotide cytosine residue is a common biochemical modification in eukaryotic DNA (193). The status of DNA methylation changed vigorously and regulated the programmed cell death during sepsis.

Binnie et al. (194) performed the first epigenome-wide methylation analysis of whole blood DNA samples from a group of severely ill patients with sepsis and non-sepsis individuals (68 patients with sepsis and 66 patients without sepsis). It was found that 668 sites of differentially methylated regions (DMRs) were associated with sepsis status. Functional enrichment analysis of 443 DMR-related genes showed that these genes encoded major histocompatibility complex class proteins, methyltransferase, and proteins involved in cell adhesion and connection. However, Feng et al. (195) found a significant difference in methylation patterns in blood DNA samples from patients with acute respiratory distress syndrome (ARDS) caused by sepsis through methylation analysis. These discrepant findings suggested a changed DNA methylome for different statues of sepsis. Morever, Lorente-Sorolla et al. (196) performed DNA methylation analysis of monocytes from patients with sepsis and healthy controls, showing significant differences in DNA methylation at CpG sites associated with monocyte-related immune responses. Most importantly, the DNA methylation profile was related to the levels of IL-10 and IL-6 and the severity of organ failure. *In vitro* experiments have shown that changes in DNA methylation of monocytes were determined by TLR stimulation as well as changes in the levels of inflammatory cytokines (196).

Referring to the methylation in TECs during sepsis, Xie et al. (197) highlighted the function of methyl-CpG binding domain protein 2 (MBD2). They showed that knockdown of MBD2 in Boston University mouse proximal tubule (BUMPT) cells or global knockout of MBD2 in mice could significantly improve survival. Gene expression analysis and chromatin immunoprecipitation analysis demonstrated that MBD2 directly binded to the CpG island of the promoter region of PKCη by inhibiting promoter methylation, resulting in the inactivation of downstream p38 MAPK and extracellular signal-regulated kinase (ERK) 1/2, and finally inhibiting the apoptosis of TECs.

#### RNA m6A Methylation

Epigenetic modification of RNA is a type of regulation at the post-transcriptional level. N6-methyladenosine (m6A) is a newly defined RNA methylation modification in eukaryotic mRNA (198, 199), affecting all basic properties of RNA, including mRNA processing, stability and translation (200), and plays various physiological roles (201–204). The m6A methylation of mRNA is dynamically regulated by a series of enzymes named “writers,” “erasers,” and “readers.” “Writer” is a methyltransferase that incorporates methyl groups into adenosine residues, “eraser” is a demethylase that removes methyl groups, and “reader” is a protein that recognizes and interacts with m6A (205–207).

Du et al. (208) reported that ablation of the m6A methyltransferase subunit methyltransferaselike 14 (METTL14, a “writers” enzyme) in myeloid cells aggravated the response of macrophages in mice to acute bacterial infections. The deletion of METTL14 diminishes m6A methylation of SOCS1 and reduces the binding of YT521-B homology (YTH) domain proteins (YTHDF1, a “reader” enzyme) to the m6A site, leading to excessive activation of TLR4/NF-κB signals. Overexpression of SOCS1 in METTL14- or YTHDF1-depleted macrophages could rescue the super-reactive phenotype of these macrophages *in vitro* and *in vivo*. Moreover, forced expression of FTO in macrophages mimics the phenotype of METTL14-deficient macrophages. Besides, METTL3 (also a “writers” enzyme) was increased after the stimulation of LPS in macrophages and the overexpression of METTL3 had anti-inflammatory effects (209). These evidence suggest that m6A statues in macrophages may have an important implication in inflammation. Recently, Shen et al. (210) evaluated the m6A profile of aortic mRNA and lncRNA in rats during LPS-induced sepsis which caused a significant downregulation of METTL3 and WTAP in aortic tissues. However, there is currently none direct evidence of the mRNA statues in endotoxin treated ECs. For TECs, Zhu et al. (211) performed m6A RNA immunoprecipitation and found the changed methylation of lncRNA MALAT1 in HK-2 cells stimulated with LPS. Pre-administration of dexmedetomidine inhibited the expression of ALKBH5 (an “erasers” enzyme) after LPS stimulation, while dysfunctional demethylation of ALKBH5 reduced the expression of MALAT1, which finally induced apoptosis and reduced the production of inflammatory factors. However, the above design was conducted *in vitro* cell models of sepsis. The systemic effects of RNA methylation should be evaluated in the standard animal model of sepsis-AKI. The function of the membranes of “writers, erasers, and readers” should be further investigated, especially their specific interactions with programmed cell death in different renal cell types including marcophages, vascular ECs and TECs.

## Conclusions

Sepsis-AKI is currently recognized as one of the most complex diseases. However, the existing treatments are mainly supportive. Therefore, it is urgent to provide new insights into the pathogenesis of sepsis-AKI and to develop effective treatment strategies. Unlike other types of AKI, the pathology of sepsis-AKI involves the participation of multiple organs and cell types. Macrophages, vascular ECs and TECs are the main cell types involved in sepsis-AKI. The programmed cell death of these cell types is precisely controlled. The necroptosis and pyroptosis of macrophage can amplify the inflammatory response systemically or locally. The dysfunction and death of vascular ECs lead to the disturbance of renal microcirculation and the leakage of activated insults from the blood. Finally, the death forms of apoptosis, necroptosis, and pyroptosis are all observed in TECs, which is the pathophysiological basics for sepsis-AKI. At present, numerous drugs targeting programmed cell death have been evaluated in treating sepsis-AKI, with the meritable intervention drugs summarized in Table 1. However, whether these drugs are effective in clinic needs further more comprehensive evaluation.

With the rapid progression in sequencing technology, metabolomics, as well as epigenomics, great advances have been made concerning the understanding of renal disease. With respect to AKI induced by sepsis, mitochondria dysfunction and oxidative stress are traditionally considered as the leading triggers of renal cell damage. Other potential regulational signals may control the fate of cells during sepsis. Mitochondria quality control, epigenetics and autophagy are the recently highlighted to regulate the damage process of sepsis-AKI. Continuing research in sepsis-AKI can faciliate the finding of sensitive and specific diagnostic biomarkers, exploring the effective therapeutic targets and developing effective remedial drugs, in order to prevent the high mortality of sepsis and the risk of sepsis-AKI.

## Author Contributions

CL, WW, X-mM, and J-gW wrote the paper. S-sX, W-xM, and YH: collected literature. Q-wF and YC produced the chart. Modified by J-nW, QY, H-dL, JJ, and M-mL. All authors participated in this review and approved the final version of the manuscript.

## Funding

This work was supported by the National Natural Science Foundation of China (Nos. 81970584 and 81800606).

## Conflict of Interest

The authors declare that the research was conducted in the absence of any commercial or financial relationships that could be construed as a potential conflict of interest.

## Publisher's Note

All claims expressed in this article are solely those of the authors and do not necessarily represent those of their affiliated organizations, or those of the publisher, the editors and the reviewers. Any product that may be evaluated in this article, or claim that may be made by its manufacturer, is not guaranteed or endorsed by the publisher.
